# Anionic Polymerization of Para-Diethynylbenzene: Synthesis of a Strictly Linear Polymer

**DOI:** 10.3390/polym14050900

**Published:** 2022-02-24

**Authors:** Vyacheslav M. Misin, Irina E. Maltseva, Alexander A. Maltsev, Alexander V. Naumkin, Mark E. Kazakov

**Affiliations:** 1 N.M. Emanuel Institute of Biochemical Physics RAS, 4 Kosygina Street, 119334 Moscow, Russia; misinnn@gmail.com (V.M.M.); sadnesscurer@gmail.com (A.A.M.); 2 A.N. Nesmeyanov Institute of Organoelement Compounds RAS, 28 Vavilova Street, 119991 Moscow, Russia; naumkin@ineos.ac.ru; 3 A.N. Frumkin Institute of Physical Chemistry and Electrochemistry RAS, 31 bld.4 Leninsky Prospect, 119071 Moscow, Russia; 4 SPC UVICOM Ltd., 5 Kolontsova St., 141009 Mytischi, Russia; mark_kazakov@mail.ru

**Keywords:** poly-*p*-diethynylbenzene, diphenyldiacetylene, anionic polymerization, n-butyllithium, heat resistance, thermal oxidation stability, stereoisomers, modification

## Abstract

Anionic homo- and copolymerization of *p*-diethynylbenzene in the presence of n-BuLi in polar solvents was carried out. The use of hexamethylphosphortriamide (HMPA) makes it possible to synthesize a completely linear soluble polymer that does not have branching and phenylene fragments. A copolymer of *p*-diethynylbenzene with diphenyldiacetylene was synthesized. Homo- and copolymers of *p*-diethynylbenzene have high thermo- and thermo-oxidative stability. By the interaction of side reactive ethynylphenylene groups with various reagents, it is proposed to synthesize clusters along the conducting chain of poly-*p*-diethynylbenzene. Due to presenting C≡CH side groups, boron, copper, and cobalt derivatives were synthesized. It is shown that not all theoretically possible stereoisomers can be formed as a result of the polymerization. The application of *p*-diethynylbenzene polymers for the modification of industrial samples of epoxy novolac resin, oligoester acrylates, and carbon fibers has been demonstrated.

## 1. Introduction

A large number of publications devoted to the synthesis and study of the physicochemical properties of substituted polyacetylenes and polydiacetylenes is explained by their unique properties such as thermo- and solvatochromism, optical characteristics, catalytic activity, and photoelectric sensitivity [1,2,3,4,5]. Homo- and copolymers of *p*-diethynylbenzene (DEB) as polyarylacetylene resins have additional properties that distinguish them in this class of polymers: good graphitization, high thermal resistance and increased coke residue, which is important when creating carbon-carbon composite materials for aviation and rocket engineering [6,7,8,9,10,11,12]. Using the DEB polymer, it was proposed to produce composites of carbon nanotubes [13] and quartz glass [14], Pt/C electrocatalysts with Pt nanoparticles in highly porous carbon spheres for fuel cells [15], humidity sensors [16], and micro-mesoporous meshes with increased adsorption capacity to hydrogen [17]. A wide variety of initiators are used for polyDEB synthesis, including quite rare and exotic ones [1,3,4,5]. In these cases, branched and/or cross-linked polymer chains are usually obtained, which may have not only vinyl links but also phenylene fragments.

This article is devoted to the homo- and copolymerization of *p*-diethynylbenzene (DEB) in the presence of *n*-Butyl Lithium (*n*-BuLi) and to the study of the structure of the obtained polymers. The advantage of using *n*-BuLi lies in its wide practical application in the industrial synthesis of liquid rubbers—precursors of adhesives and sealants. In addition, the processes of modification of the obtained polymers were carried out, as a result of which Cu, Co, B heteroatoms were introduced into the polymers. Previously, a very short first report was published on the possibility of DEB polymerization in the presence of this catalyst [18]. In it, the fundamental possibility of synthesizing a soluble DEB homopolymer with a previously unknown linear structure was reported.

## 2. Materials and Methods

### 2.1. Materials

Argon was purified by passing through three consecutive columns filled with a chromium-nickel catalyst (one column with operating temperature 180 °C) and 4 Å molecular sieves (two columns with room operating temperature).

Phenylacetylene (PA, 98%, Aldrich, St. Louis, MO, USA) was distilled under vacuum at 37.5 °C/2.0 kPa. 1,4-diethynylbenzene (pDEB, 95%, Aldrich, St. Louis, MO, USA) was recrystallized from hexane and sublimated.

N-butyl lithium (*n*-BuLi) (1.6 M in hexane, Aldrich, St. Louis, MO, USA) was poured into thin-walled spherical glass ampoules with volume 0.3–1.0 mL by a special vacuum technique.

Decaborane (98%, Alfa Aesar, Tewksbury, MA, USA), N, N-dimethylaniline (99.5%, Aldrich), dicobaltoctacarbonyl (Co_2_(CO)_8_, 98%, Aldrich) were all used as received.

1,4-diphenylbutadiyne (diphenyldiacetylene, DPDA) [19] and 1,4-diphenylbuten-1-yne-3 [20] were prepared according to the literature.

Dimethyl sulfoxide (DMSO, 99%, Aldrich, MO, USA), hexamethylphosphoramide (HMPA, 97%, Acros Organics, Waltham, MA, USA), and tetrahydrofuran (THF, 99.0%, Aldrich, MO, USA) were dried by 3 Å/4 Å molecular sieves before use. Other solvents, including benzene (99%, Aldrich), toluene (99%, EMPLURA, Darmstadt, Germany), hexane (95%, Aldrich), and N, N-dimethylformamide (DMFA, 99%, EMPLURA, Darmstadt, Germany), were all used as received.

The hydrochloric acid (37% in water, Aldrich), ammonium hydroxide (28%, Aldrich), hydroxylamine hydrochloride (96%, Merck, Kenilworth, NJ, USA), and KBr (ACS reagent, Aldrich) were all used as received.

The CuCl salt after long-term storage was washed with water acidified with hydrochloric acid until the blue color in the washing water ceased to appear. Next, the salt was washed twice with acetone and dried under a vacuum. The resulting white product was stored under argon in a tightly sealed vessel.

To study the modification of industrial materials, we used oligoester acrylates triethyleneglycoldimethacrylate (TGM-3), bis-(methacryloylethylenecarbonate)diethylene-glycol (OCM-2) and epoxy novolac resin EN-6 (condensation products of epichlorohydrin with novolac phenol-formaldehyde resin SF-0113, analog DEN-438).

### 2.2. Measurements

The weighted average molecular mass (${\bar{M}}_{w}$), polydispersity index (${\bar{M}}_{w}/{\bar{M}}_{n}$) and molecular mass distribution of the polymers were measured on a Waters GPC Model 208 chromatograph at 25 °C, using THF as the eluent (1 mL/min) and standard polystyrene as the reference.

Fourier transform infrared (FT-IR) spectra were recorded on a Thermo Scientific Nicolet 6700 Analytical FTIR spectrometer (Thermo Fisher Scientific, Waltham, MA, USA). All samples were prepared as KBr pellets.

^1^H NMR and ^13^C NMR spectrum were recorded in CDCl_3_ solution on a Bruker Avance III 500 NMR spectrometer, using tetramethylsilane as internal standard, frequency 500.18 MHz (^1^H); 125.78 MHz (^13^C).

Thermal gravimetry and differential thermal analysis curves were simultaneously recorded with a Stanton 801 STA Thermoanalyzer (Netzsch, Germany), using Pt crucibles, Pt/(Pt, 13% Rh) thermocouples and heating rates of 5 °K·min^−1^.

Thermal oxidation stability of carbon fiber was determined by thermovolumetry on a manometric installation [21]. It allowed measuring the kinetics of O_2_ absorption (oxygen pressure 20 kPa) and quantifying the mass loss of the sample at 600 °C for 60 min.

The results of measuring the electrical resistivity were obtained with the four-probe method as in [22].

X-ray photoelectron spectra were acquired with a VIEE-15 spectrometer (Varian, US) using Al K_α_ (1486.6 eV) radiation at an operating power of 150 W of the X-ray tube. Survey and high-resolution spectra of appropriate core levels were recorded at pass energies of 160 and 40 eV and with step sizes of 1 and 0.1 eV, respectively. The samples were mounted on a sample holder with a two-sided adhesive tape, and the spectra were collected at room temperature. The base pressure in the analytical UHV chamber of the spectrometer during measurements did not exceed 1.33 × 10^−6^ Pa. The energy scale of the spectrometer was calibrated to provide the following values for reference samples (i.e., metal surfaces freshly cleaned by ion bombardment): Au 4f_7/2_–83.96 eV, Cu 2p_3/2_–932.62 eV, Ag 3d_5/2_–368.21 eV. The surface charge was taken into account according to the C–C/C–H state identified in the C 1s spectrum, to which a binding energy of 285.0 eV was assigned. After charge referencing, a Shirley-type background with inelastic losses was subtracted from the high-resolution spectra.

Elemental analyses were made by the Microanalysis Laboratory, INEOS RAS, Moscow, Russia.

### 2.3. Polymerization

DEB polymerization in the presence of *n*-BuLi was carried out in a four-neck reactor equipped with a thermostatically controlled jacket, a stirrer, a thermometer, an Ar insertion tube, and a special funnel in which a spherical microampule with *n*-BuLi was broken. Previously, all the glassware was thoroughly washed, calcined at a temperature of 200 °C for three hours, and cooled in the Ar current. A monomer and a calculated amount of solvent were loaded into the reactor purged with Ar and the resulting solution was heated to 55 °C. A spherical microampule with *n*-BuLi ([*M*]_0_/[*I*]_0_ = 15) was placed in the funnel and broken with a glass striker. The initiator remainders were washed off with 1–2 mL of solvent. The funnel and reactor were purged with dry argon during polymerization. The beginning of the reaction was accompanied by the staining of the solution in intense blue (HMPA) or red (DMSO), then the color changed to blue-green. After the expiration of the reaction time, 1–2 mL of water was added to the reaction mixture to destroy the active centers. The reaction mixture was planted in a tenfold excess of 2% HCl. The precipitate was washed with distilled water and dissolved in benzene. The insoluble benzene fraction was separated on the filter and the reaction mixture was planted in a tenfold volume of hexane or ethyl alcohol. The reaction mixture for analysis by the gel-permeation chromatography (GPC) was selected with a washed, dried, and argon-purged pipette. The yield of the PDEBA polymer was determined gravimetrically. Copolymers were synthesized in a similar way.

### 2.4. Synthesis of polyDEB π-Complexes with Co_2_(CO)_8_

A 10% solution in benzene of the calculated amount of Co_2_(CO)_8_ was added drop by drop to the polyDEB solution in benzene while stirring. The reaction mixture was stirred at room temperature until the release of CO was completely stopped and Co_2_(CO)_8_ disappeared in the reaction mixture. The presence of Co_2_(CO)_8_ in the reaction mixture was determined by thin-layer chromatography in hexane. After the end of the reaction, the reaction mixture was filtered through a filter and planted in a tenfold excess of hexane. The precipitate was dried in a vacuum at 30 °C.

### 2.5. Synthesis of Polymeric σ-Acetylides of Copper

An equal volume of freshly distilled THF was added to the CuCl solution in 25% NH_4_OH with a small amount of hydroxylamine hydrochloride. With stirring, polyDEB solution into THF was poured. Three hours later the reaction mixture was planted in a tenfold excess of 15% ammonia water solution. The precipitate was washed with water, alcohol and benzene, and dried in a vacuum.

### 2.6. Synthesis of Carborane-Containing Polymers DEB

To the polyDEB or CPA solution in toluene, a toluene solution of decaborane (0.5 or 1 mol B_10_H_14_ per 1 mol –C≡CH) and 2 moles of N, N-dimethylaniline were added. The solution was stirred for 8 h at 90 °C until the release of H_2_ was completely stopped. The toluene solution was washed with hydrochloric acid, distilled water and planted in a tenfold excess of hexane.

## 3. Results

### 3.1. Synthesis and Characterization of Homo-Polymers

The electrophilicity of the C≡C bond in DEB, as well as the electron acceptor character of the second substituent, Ph–C≡CH, suggested that DEB would be sensitive to the action of nucleophilic agents [1]. For example, anionic polymerization of phenyl-containing diphenylacetylene and diphenyldiacetylene (DPDA) [23] was effective. Thus, DEB polymerization had to be initiated by anionic initiators. The synthesis of soluble polyDEB in the presence of *n*-BuLi in the polar solvents DMSO and HMPA has been reported [18,24]. The polymers were yellow-brown powders, soluble in aromatic and chlorinated hydrocarbons, ketones, DMFA, HMPA, DMSO, and insoluble in alcohols and alkanes. The dependence of the yield and properties of linear polyDEB on the conditions of anionic polymerization are shown in Table 1.

**Figure 1:**
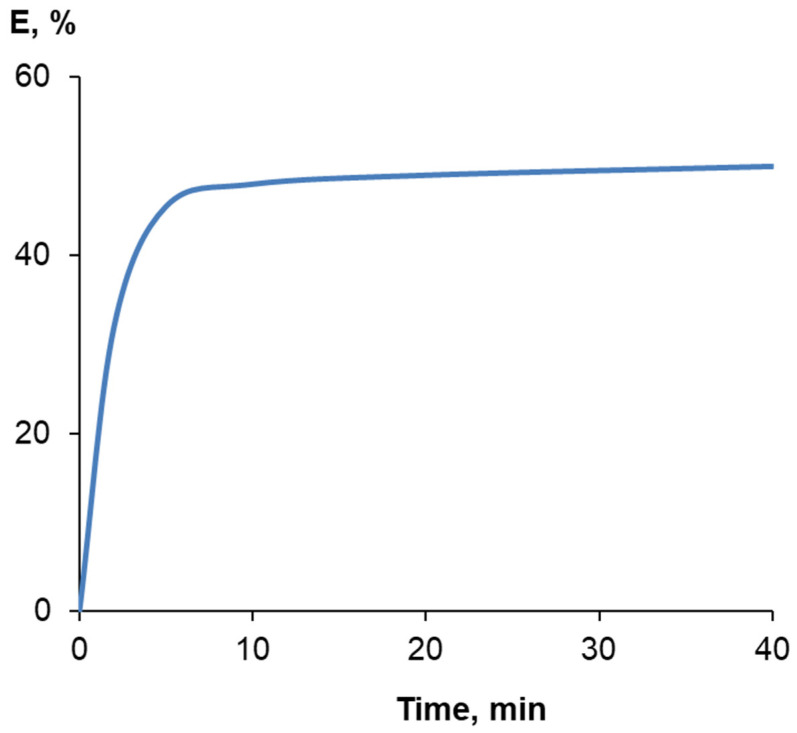
Dependence of PDEBA yield on polymerization time (HMPA, *n*-BuLi, t = 55 °C, [*M*]_0_/[*I*]_0_ = 15:1).

Figure 1 shows the kinetic curve of DEB polymerization in an HMPA medium. It can be seen that polymerization begins without an induction period and proceeds at a constant rate, which decreases when a certain conversion is achieved, and then the process almost completely stops. The reason for the termination of polymerization can be caused by the exchange interaction of the active center with the π-electron system of the growing chain (electron delocalization) [25] or the polymer-monomer (donor-acceptor) interaction of the components of the reaction system [25,26]. These possible causes were considered by the authors during the anionic polymerization of phenylacetylene (structural analog of DEB) in the presence of lithium organic compounds.

**Figure 2:**
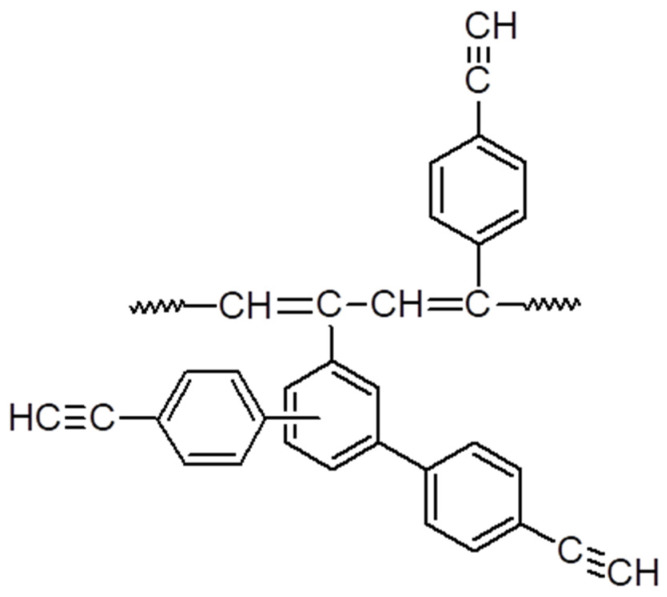
Possible variants of the intramolecular structure of the PDEBA.

PDEBA polymer chains, in principle, can have linear polyene and phenylene fragments (Figure 2). To determination the intramolecular structure of polymers, their NMR and IR spectra were investigated.

**Figure 3:**
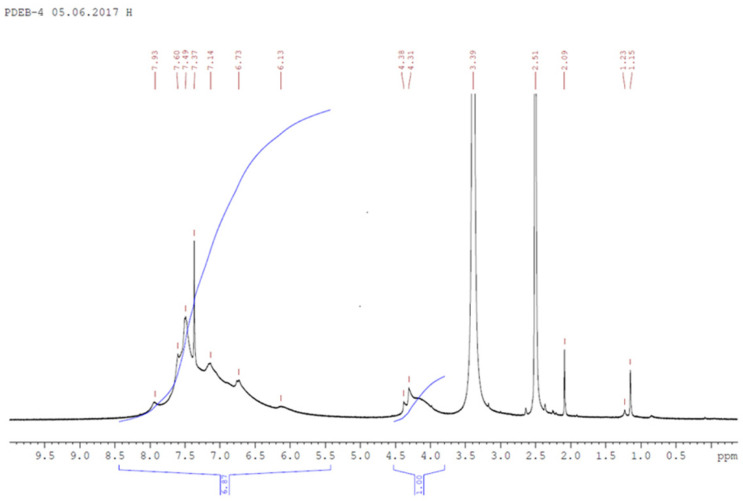
^1^H NMR spectrum PDEBA-4. (acetone-D6).

In the ^1^H NMR spectrum of a polymer obtained in an HMPA medium (Figure 3), the signals of protons of phenyl nuclei and protons with a double bond of the polymer chain are manifested by a complex, poorly resolved multiplet in the range δ = 6.2–8.3 ppm. The signals of ethynyl protons are observed as a widened singlet line at 3.6 ppm. The ratio of the sum of the integral intensities of the signals of aromatic and olefin protons to the integral intensity of the signal of protons of ethynyl groups, for polymers obtained in HMPA, is 5:1, which corresponds to the presence of one ethynyl group in the elementary unit of the macromolecule (if we assume a polyene structure in the polymer). Polymers obtained in DMSO have similar ^1^H NMR spectra; however, the ratio of the integral signal intensities of aromatic and olefin protons to ethynyl protons for them is 7:1. This can be explained by the partial disclosure of the second ethynyl group in DEB with the appearance of branching.

In all spectra, there are signals in the range δ = 1.1–1.3 ppm of terminal butyl groups formed due to the attachment of the initiator to DEB. However, calculations carried out taking into account the molecular weights of polymers demonstrate a deficiency of Bu-groups.

**Figure 4:**
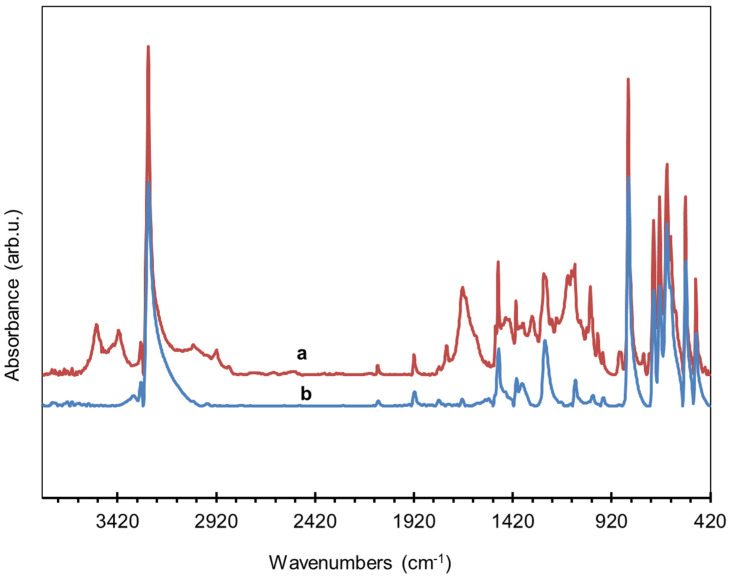
IR spectra of PDEBA-5 (a) and p-DEB (b).

In the IR spectrum of PDEBA (Figure 4), the presence of free ethynyl groups in the polymer is confirmed by the preservation of a strong peak of 3300 cm^−1^ (valence vibrations ≡C–H) and a weak peak at 2100 cm^−1^ (valence vibrations C≡CH) [27]. A decrease in the intensity of the peaks of valence vibrations associated with triple bonds and the appearance of peaks at 3040 cm^−1^ (valence vibrations =CH), a wide band at 1680 cm^−1^ (valence vibrations of trans C=C bonds), and a weak peak at 990 cm^−1^ (deformation vibrations of trans C=C bonds) indicate that polymerization has taken place with the opening of a part of triple bonds and the formation of a polyene chain.

**Figure 5:**
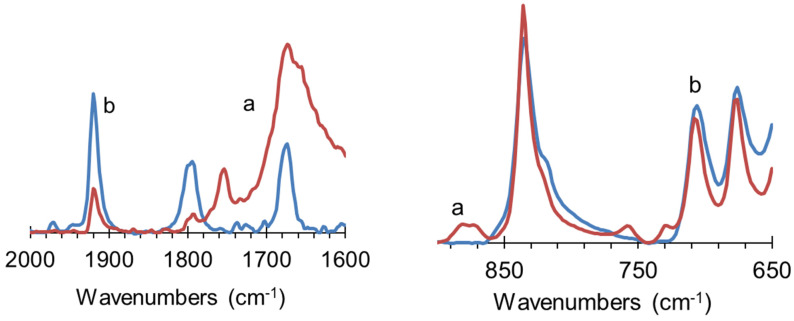
Increased fragments of PDEBA-5 (a) and p-DEB (b) IR spectra.

To determine the type of substitution of benzene rings, IR spectra of 10% polymer and monomer solutions in the regions of 2000–1650 cm^−1^ and 900–650 cm^−1^ were measured. The peaks of the IR spectra of polymers have predominantly the same frequencies as the corresponding peaks in the monomer (Figure 5). The formation of micropeaks at 726, 760, and 880 cm^−1^ can be caused by both out-of-plane deformation vibrations of the polyene chain and partial trimerization of the monomer. The appearance of a peak at 1756 cm^−1^ in the region of compound frequencies and overtones (2000–1650 cm^−1^) may be caused by the presence of micro-quantities of 1,3,5-substituted phenylene fragments.

However, the use of anionic initiators is not accompanied by the formation of cyclic trimers from substituted acetylenes, as is observed in the case of organometallic complexes and metal salts [1,5,28].

Nevertheless, the possibility of the occurrence of 1,3,5-substituted phenylene fragments having a band in the region of 881 cm^−1^ was considered [29]. The absence of such a band proved the absence of 1,3,5-substituted phenylene fragments in PDEBA. In addition, there is no peak in the 900–860 cm^−1^ region of the IR spectrum of the polymer, which corresponds to out-of-plane deformation vibrations of the C–H bond of tetra-substituted benzene. Thus, consideration of the 2000–1650 and 900–860 cm^−1^ regions allows us to conclude that the formation of a noticeable number of tri- and tetra-substituted phenylene fragments does not occur.

In addition, there are bands in the IR spectrum for which the terminal butyl group is responsible (2920 cm^−1^ valence, 1400–1300 cm^−1^ deformation vibrations of the C–H bond). The presence of a terminal butyl group in the polymer according to ^1^H NMR and IR spectroscopy data suggests that the initiation of polymerization occurs with the opening of the triple bond, the addition of the butyl group to the monomer, and the formation of a carbanion. Such polymerization is classical in the anionic polymerization of vinyl, diene [30], and acetylene [1] monomers.

**Scheme 1:**
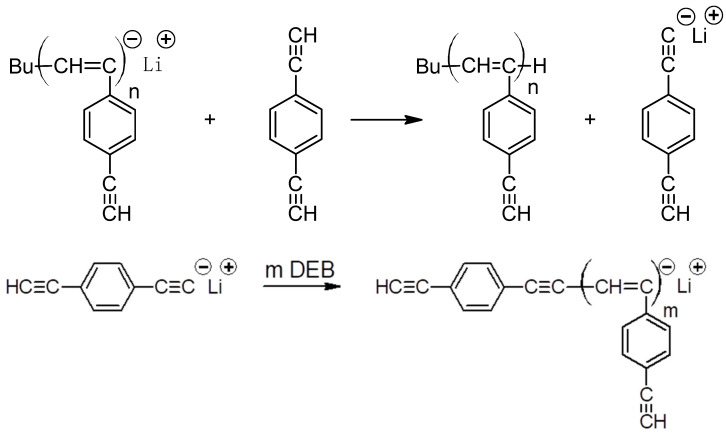
Chain transfer reaction to DEB according to [31].

Interestingly, a weak band of 2190 cm^−1^ was detected in the IR spectrum of polymers, which is characteristic of the valence oscillation of the di-substituted bond –C≡C–. The appearance of this band could be explained by the fact that the chain is transferred to the monomer during polymerization, as was observed in the case of the polymerization of phenylacetylene (Scheme 1) [31]. At the same time, the H_ar+ol_/H_eth_ ratio should increase, which was observed in reality.

However, the GPC results contradict this assumption. It can be seen (Table 2) that there is no decrease in the experimental average calculated degree of polymerization ${\bar{n}}_{exp}$

(1) $${\bar{n}}_{exp}=\frac{{\bar{M}}_{n}}{MM_{DEB}}\;$$

where *MM_DEB_* is the molecular mass of DEB.

Compared to the theoretical ${\bar{n}}_{theor}$, calculated for a process going without chain transfer to a monomer

(2) $${\bar{n}}_{theor}=\frac{{\left[M\right]}_{0}\cdot P}{{\left[I\right]}_{0}}$$

where [*M*]_0_ and [*I*]_0_ are the initial concentrations of the monomer and initiator, P is the polymer yield.

**Scheme 2:**
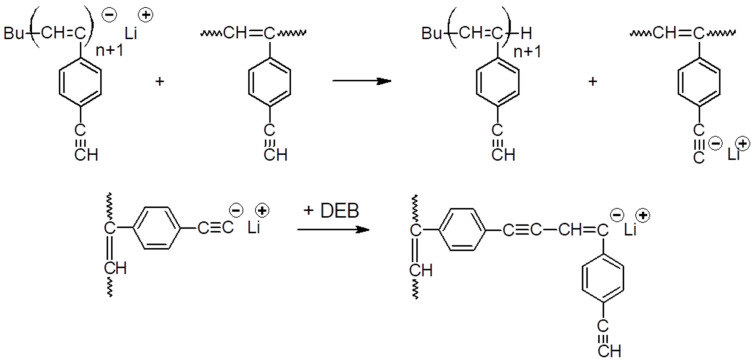
The reaction of the chain transfer to the polymer.

The discrepancy between the results of IR spectroscopy (the presence of a peak of 2190 cm^−1^) and GPC can be explained by assuming that the chain is transferred to the polymer due to proton migration from the side substituent –PhC≡CH to the polymer carbanion. In this case, branching occurs at the main chain and the corresponding disubstituted C≡C bonds in the polymer chain (Scheme 2).

The lateral phenylene-ethynyl carbanion ~CH=C(Ph–C≡C^−^ Li^+^)~ formed during the chain transfer reaction to the polymer is similar in structure and identical in properties to lithium phenylacetylide Ph–C≡C^−^ Li^+^. It is known that lithium phenylacetylide is capable of initiating polymerization under these conditions [25,31]. These facts indicate the possibility of a chain transfer reaction to the polymer according to Scheme 2.

**Figure 6:**
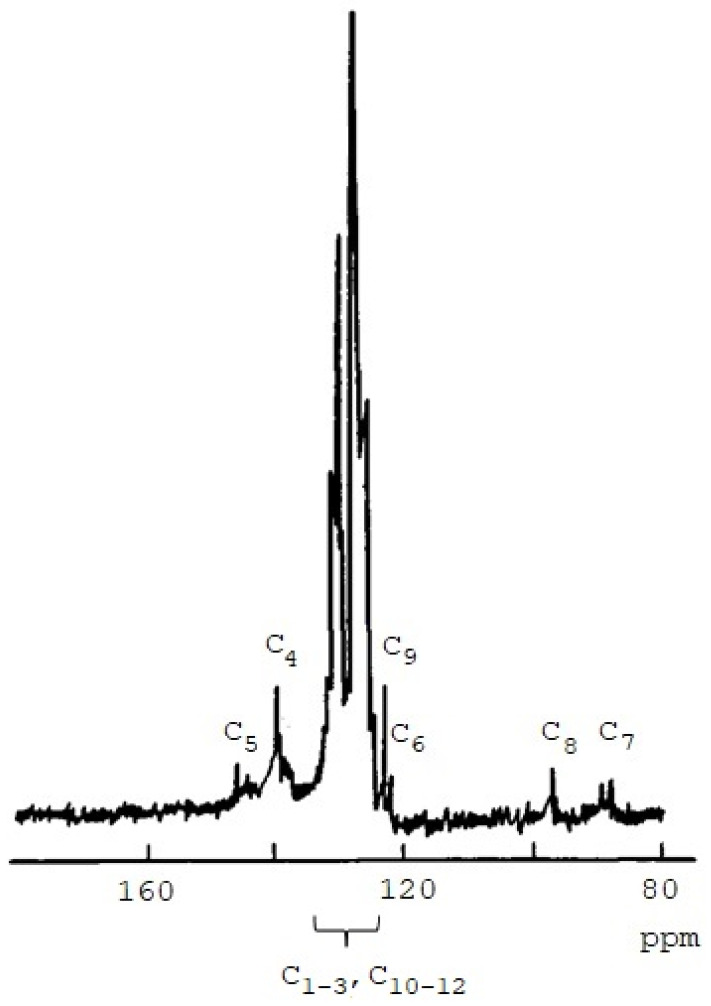
^13^C NMR spectrum of 1,4-diphenylbutene-1-yn-3.

To confirm the above-described structural features of PDEBA, a low-molecular trans-1,4-diphenylbutene-1-in-3 (**I**) was synthesized, which models the polymer link and the emerging active center quite well. Its ^13^C NMR spectrum (Figure 6, Table 3) was compared with that of PDEBA.

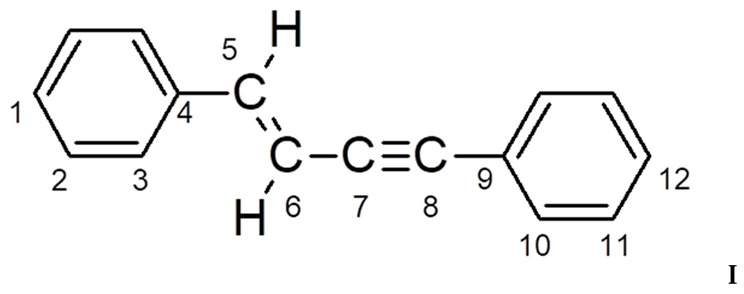



**Table 3:** Values of chemical shifts δ in the ^13^C NMR spectrum of 1,4-diphenylbutene-1-yn-3.

Atom C	Chemical Shifts δ, ppm	
	Theory [32]	Experiment
C_1_	127.7	128.1
C_2_	128.4	128.6
C_3_	126.2	126.3
C_4_	137.4	136.4
C_5_	141.7	141.3
C_6_	106.3	108.2
C_7_	88.5	89.0
C_8_	95.5	91.8
C_9_	122.3	123.5
C_10_	132.1	131.5
C_11_	128.1	128.7
C_12_	128.2	128.2

In the ^13^C NMR spectrum of the polymer, there are signals δ = 83.56 and 83.38 ppm of the C≡CH group. There are signals of carbon at the double bond of the polyene chain with δ = 140.62 and 121.80 ppm. Similar signals with δ = 142.8 and 126.6 ppm were observed in [33] for carbons of the C=C bond of trans-polyphenylacetylene. In addition, there is a signal of carbon =Ĉ(Ph)– with δ = 140.62 ppm. A similar signal has a phenyl-substituted polyene homopolymer DPDA synthesized in the presence of [Co_2_(CO)_6_]_2_·PhC≡C–C≡CPh, (δ = 144.2 ppm) [34] and carbon C_5_ in I. The signal in the polymer at δ =109.38 ppm is similar to that of the C_6_ atom (δ = 108.215 ppm) of Model **I** (Table 3). Thus, ^13^C NMR spectroscopy confirms the existence of fragments (**II**) with C≡C groups in the PDEB and the transfer of the chain to the polymer. The fact of chain transfer to the polymer explains the reason for the deficiency of Bu groups in polymer chains according to the results of ^1^H NMR spectra.

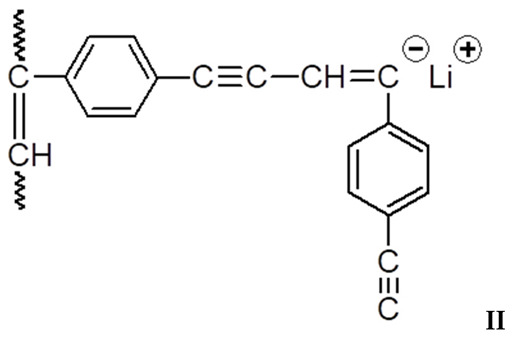



The intensity of the chain transfer process to the polymer increases during the transition from HMPA to DMSO and with increasing polymerization time, as can be seen from the decrease in the number of triple bonds per macromolecule link (Table 1). This is confirmed by the results of GPC: during the transition from HMPA to DMSO and with an increase in polymerization time, the values of ${\bar{M}}_{w}$ and ${\bar{M}}_{z}$ increase (the proportion of relatively high molecular fraction increases), and the ratios of ${\bar{M}}_{w}/{\bar{M}}_{n}$ and ${\bar{M}}_{z}/{\bar{M}}_{w}$ increase (the molecular mass distribution widens) (Table 4). Comparison of the results, which was shown in Figure 1 and Table 4, allows us to say that after 10% conversion of DEB, an inefficient process of monomer transformation occurs due to the formation and lengthening of branches in the polymer molecule.

The course of DEB polymerization with selective disclosure of only one ethynyl bond and a decrease in the reactivity of the remaining unreacted ethynyl group in PDEBA compared to DEB can be explained as follows. One of the C≡CH groups in monomeric DEB is conjugated with a phenylethynyl substituent HC≡C–Ph–, which is a strong electron acceptor. Therefore, the –C≡CH group in the monomer polymerizes well in the presence of anionic initiators [1,30]. Since there is no conjugation in the polymer between the polyene chain and the substituent H–C≡C–Ph– [35], the free ethynyl group in the side substituent PDEB is conjugated only with the phenyl ring. This ring has weaker electron acceptor properties than the ethynylphenylene group of the initial monomer conjugated with another polymerizing ethynyl group. Consequently, the reactivity of the remaining ethynyl group in the PDEBA side substituent in the polymerization reaction will be less compared to the reactivity of the ethynyl group in the monomer.

The results obtained suggest that DEB polymerization in the presence of *n*-BuLi can take place according to the classical single-center anionic mechanism [30] with initiation due to the addition of *n*-BuLi to the monomer. As a result, polymers with links of trans-polyene structure 3 are formed, in concordance with Scheme 2. In the case of the use of HMPA, only one triple bond is disclosed. In this case, the most likely connection of links is only by the head-tail type, since the active center –CH=C(PhC≡CH)^−^Li^+^ is more advantageous for anionic polymerization than –C(PhC≡CH)=CH^−^Li^+^ [1,30].

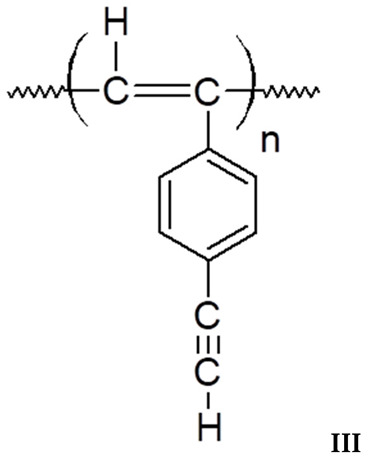

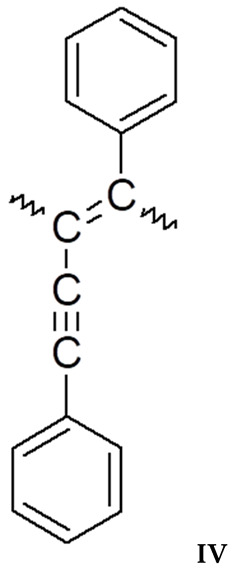





During polymerization, the chain can also be transferred to the polymer with the appearance of a C≡C bond inside the polymer chain and the appearance of branches in the polymer chain. If the polarity of the reaction medium decreases or the polymerization time increases, this process increases. An increase in the number of branches naturally leads to intermolecular crosslinking and the appearance of an insoluble fraction.

### 3.2. Synthesis and Characterization of DEB-DPDA Copolymers

Copolymers of DEB and DPDA (CPA) are paramagnetic amorphous yellow-brown powders with ${\bar{M}}_{n}$ up to 1900, soluble in acetone, benzene, DMFA, and insoluble in alcohols and alkanes. Unlike the PDEBA homopolymer, they are highly soluble in CHCl_3_. In the ^1^H NMR spectra, there are signals of protons of phenyl nuclei and protons with a double bond in the region of 6.2–8.3 ppm, signals of ethynyl protons with δ = 3.15 ppm, and signals of protons of the terminal butyl group in the region of 0.8–1.8 ppm. Polymerization conditions, yields and molecular masses are shown in Table 5.

With an increase in the proportion of DEB in the composition of the initial mixture, the number of ethynyl groups in CPA increases, as well as the yield and ${\bar{M}}_{n}$. Increasing the polymerization time reduces the number of ethynyl groups in CPA, and increases ${\bar{M}}_{n}$ and CPA yield. The latter can be explained by the reaction of free ethynyl groups, i.e., branching of macromolecules.

**Figure 7:**
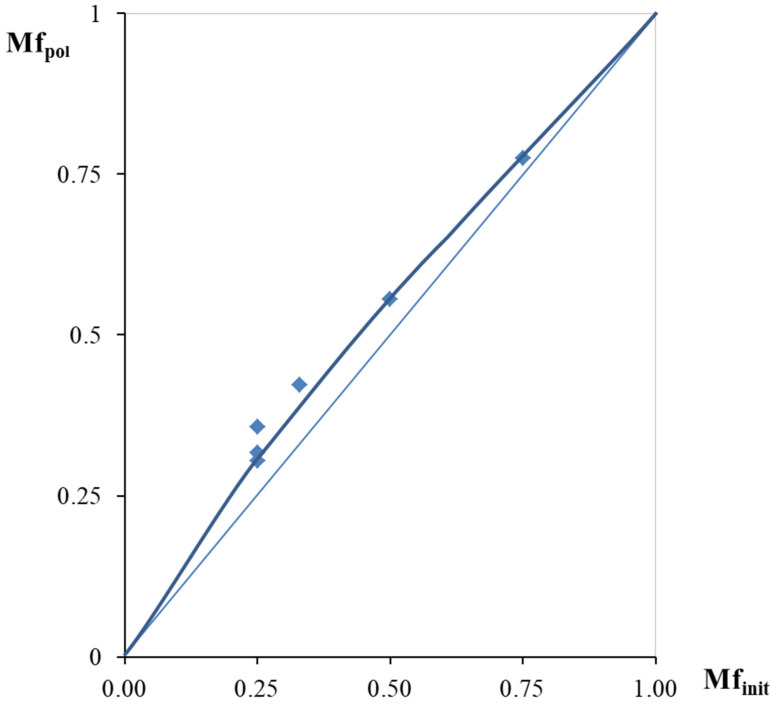
Dependence of the molar fraction of DEB in CPA Mf_pol_ on the molar fraction of DEB in the initial mixture Mf_init_.

The composition of CPA was determined by ^1^H NMR spectra. Since copolymerization was carried out under the same conditions as DEB homopolymerization in DMSO, there may be a certain number of branched links in the CPA. In addition, it was naturally assumed that the reactivity of the second ethynyl group of DEB in CPA is approximately the same as in the homopolymer. Consequently, in an equimolar mixture of monomers, the ratio of the integral signal intensities of ethynyl protons to the sum of the integral signal intensities of aromatic and olefinic protons from DEB units will be 1:7. Using these assumptions, it is possible to calculate the ratio of the integral intensities of the signals of ethynyl protons DEB to the integral intensity of the signals of aromatic protons DPDA only. This statement is true only for short reaction times since during homopolymerization of DEB, an insoluble fraction appears over time and it becomes impossible to determine exactly the number of ethynyl groups and, accordingly, the ratio of protons. CPA was prepared by copolymerization of monomers to a conversion not exceeding 4.6%. Polymerization of DEB and DPDA under these conditions proceeds mainly by one triple bond (**III**, **IV**). The diagram of the composition of the copolymer is shown in Figure 7.

Copolymerization parameters were determined using the Fineman and Ross equation for monomers with one reactive group [36].

(3) $$\frac{F\cdot \left(f-1\right)}{f}=r_{1}\cdot \frac{F^{2}}{f}-r_{2}$$

where *F* is the ratio of monomers in the initial composition, *f* is the ratio of DEB and DPDA in polymer composition, *r*_1_ and *r*_2_ are copolymerization constants of DEB and DPDA, respectively.

The results of measurements and calculations are given in Table 6.

The values of the effective copolymerization constants calculated by the least-squares method are r_1_ = 1.103 ± 0.041, r_2_ = 0.607 ± 0.038 (deviation 0.67576 × 10^−2^; correlation coefficient 0.9970). As follows from the values of copolymerization constants, the triple bond in DEB is more reactive than in DPDA.

**Figure 8:**
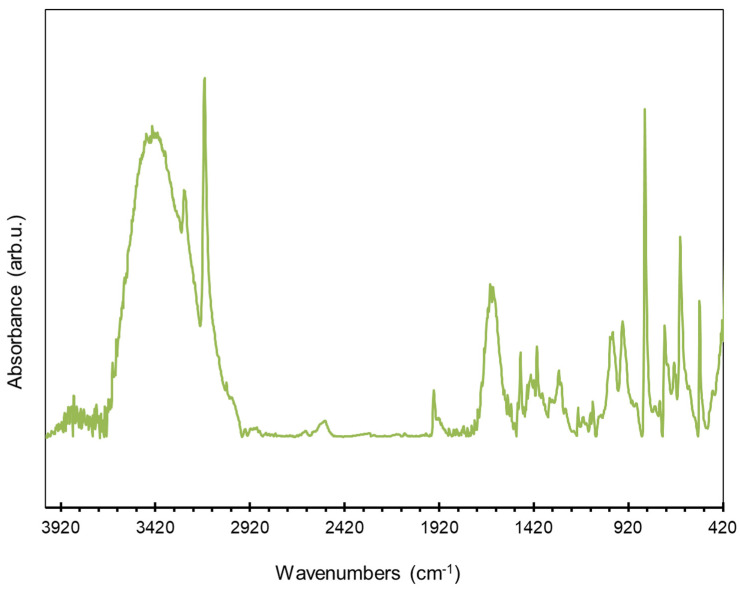
IR spectrum CPA-6.

In the IR spectrum of CPA (Figure 8) there are frequencies also observed in the spectra of PDEBA and polyDPDA homopolymers synthesized in the presence of *n*-BuLi. In particular, there is a very weak band at 2100 cm^−1^ due to valence fluctuations of the disubstituted C≡C bond. Such a triple bond is present in the polymer spectrum during DPDA polymerization by a single triple bond [23,34]. The spectrum contains signals of double bonds of 1650 cm^−1^ (valence vibrations of trans –C=C bonds) and a series of bands in the region of 1000–800 cm^−1^ (deformation vibrations of trans –C=C bonds), which indicates the formation of a polyene chain.

The overlap of peaks in the region of compound frequencies and overtones (2000–1650 cm^−1^) does not allow us to determine the type of substitution of benzene rings.

We assessed the possible presence of cumulene **V** and enyne **VI** links, the occurrence of which is still unlikely from DPDA. The formation of these links is known only in the case of solid-phase topochemical polymerization of various disubstituted diacetylenes [2].

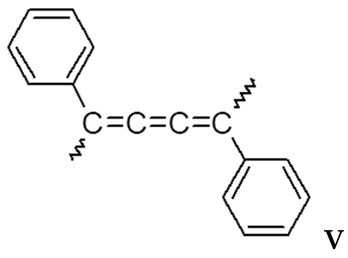

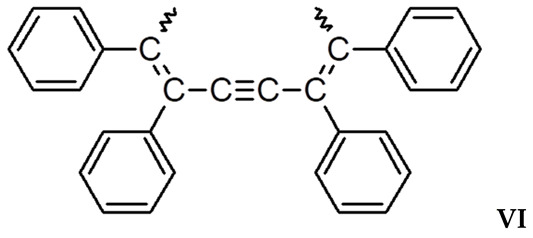





The cumulene structure **V** is possible due to the presence of a peak at 1950 cm^−1^, characteristic of cumulenes ~RC=C=C=CR~, where R= –(CH_2_)_4_OC(O)NHC_6_H_5_ [37]. At the same time, there is no strong peak of out-of-plane deformation cumulene vibration at 850 cm^−1^. To clarify the structure of the links, the ^13^C NMR spectra of CPA and the DPDA homopolymer obtained using *n*-BuLi under the same conditions as the copolymer (DMSO, T = 55 °C) were taken.

In the spectrum of anionic polyDPDA, there is a wide signal of aromatic carbon atoms in the region of 124.5–135.0 ppm. The signals δ = 97.97 and 89.38 ppm correspond to two nonequivalent carbon atoms of a triple bond. In the link of the polyenyne structure **VI**, the carbon C≡C atoms must be equivalent, therefore, there are no links of the polyenyne structure **VI** in the polymer. There are no cumulene carbon signals in the spectrum in the region of 140–180 ppm [38]. Therefore, there are no links of structure **V**. In the ^13^C NMR spectrum of anionic CPA, there are no cumulene carbon signals, there is a wide signal of aromatic carbon atoms in the region of 122–135 ppm. Carbon atoms with a triple bond from the polyene link of the DPDA give signals with δ = 99.33 and 89.94 ppm. Signals with δ = 84.12 and 83.42 ppm belong to carbon atoms of the –C≡CH group in DEB. Similar signals were observed in the ^13^C NMR spectrum of the homopolymer DEB (δ = 83.56 and 83.38 ppm). In addition, in the CPA spectrum in the region of 14–23 ppm, there are signals of carbon atoms of the terminal butyl group. ^13^C NMR spectroscopy confirms the assumption that the links in CPA have the same structure as in homopolymers.

Thus, as a result of copolymerization of DEB and DPDA in a DMSO medium initiated by *n*-BuLi, a CPA with free ethynyl groups is obtained. It consists of DEB units (**III**), polymerized by one triple bond, and DPDA units (**IV**), having the structure of a substituted polyene.

### 3.3. Investigation of PDEBA and CPA Thermal Degradation

**Figure 9:**
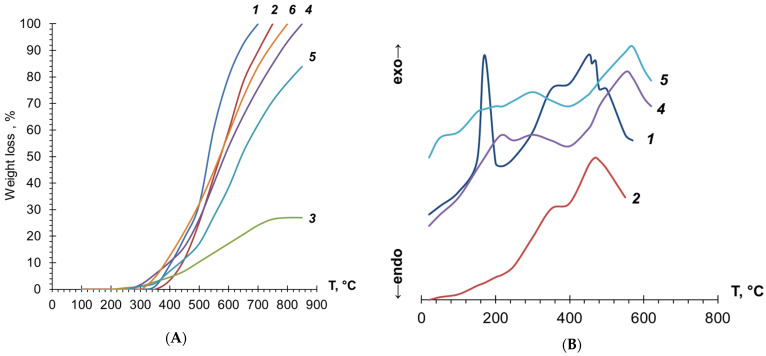
TGA (**A**) and DTA (**B**) curves, air: (**1**)—PDEBA-2; (**2**)—PDEBA-2-200; (**3**)—PDEBA-2 (vacuum); (**4**)—CPA-2; (**5**)—CPA-2-200; (**6**)—CPA-6.

The investigation of thermal degradation of PDEBA in a vacuum showed that the mass loss is equal to 20% at 700 °C (Figure 9). The mass loss at low temperatures (more than 130 °C) is associated with the loss of sorbed moisture and, possibly, with precipitator residues. As the temperature increases, a decrease in the rate of mass losses and the formation of a significant “coke” residue was noted. PDEBA and CPA are highly thermo-oxidative-resistant: no noticeable mass loss occurs up to a temperature of 400 °C (Figure 9). Further, the rate of thermo-oxidative destruction increases and the samples completely burn at temperatures of 800–900 °C. PDEBA and CPA have approximately the same thermal oxidation resistance. The temperatures of the 40% mass lost for PDEBA and copolymers with a higher (CPA-6) and lower (CPA-2) content of ethynyl groups are 520, 530, and 550 °C, respectively. It can be seen that an increase in the content of DPDA links slightly increases the thermo-oxidative stability.

On the DTA curve of the PDEBA homopolymer (Figure 9), there is an exothermic peak in the region of 175 °C associated with the opening of the bonds of C≡C by ethynyl groups. To verify the correct interpretation of this peak, PDEBA-2 thermolysis was performed in a vacuum for 6 h at 100 °C (PDEBA-2-100) and 200 °C (PDEBA-2-200). PDEBA-2-100 retained its color and partially lost its solubility in organic solvents. The number of free ethynyl groups in it according to IR spectrum data (peak 3300 cm^−1^) decreased slightly. PDEBA-2-200 has turned into a brown, insoluble in organic solvents powder. There were no free ethynyl groups in the polymer (there is no peak of 3300 cm^−1^). There was no heat release in the 175 °C region on the curve DTA PDEBA-2-200.

Similar exothermic peaks in the 200 °C area caused by the opening of the –C≡CH bonds are present on the DTA curves of CPA. The thermolysis of copolymers CPA-2 and CPA-6 (200 °C, 6 h, vacuum) leads to the formation of yellow-brown insoluble products (CPA-2-200 and CPA-6-200), which, according to IR spectrum data, do not have free ethynyl groups. There is no exothermic peak in the 200 °C area on the DTA curves of thermalized copolymers (Figure 9). The displacement of the exothermic peak on the DTA curves of copolymers to the region of higher temperatures compared to the non-thermalized homopolymer p-DEB can be explained by steric difficulties arising in copolymers due to the presence of high-volume DPDA units in them. According to the results of TGA, the resistance to thermal-oxidative degradation of PDEBA and CPA is enhanced as a result of preliminary thermolysis.

### 3.4. Steric Features of Linear PDEBA Macromolecules

PDEBA has reactive side –PhC≡CH groups. Therefore, on its basis, in principle, macromolecular acetylides, carboranes, arencarbonyl π-complexes, grafted copolymers, and other derivatives can be synthesized. Some syntheses were briefly reported in [39,40]. However, the derivatives can be synthesized only by taking into account the actual steric availability of phenylene and ethynyl fragments in polymers. Therefore, it is necessary to determine the probable structural isomers in PDEBA, taking into account the possibility of further modification of PDEBA by various fragments with heteroatoms. At the same time, only the linear structure of the PDEBA chains, in principle, can allow the creation of clusters along the conductive polymer chain.

**Figure 10:**
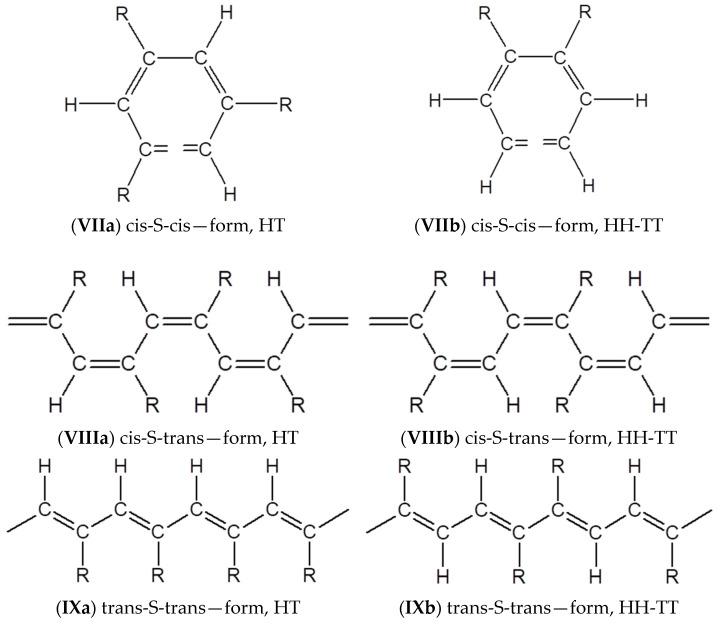

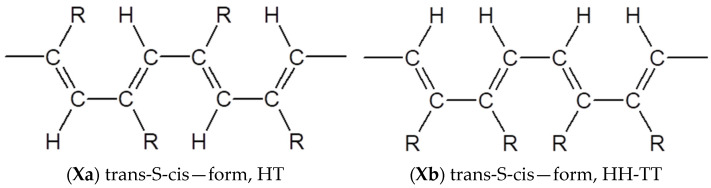
Possible conformal structures of disubstituted polyenes, where R = –PhC≡CH.

The conditions of synthesis of substituted polyacetylenes significantly affect the intramolecular structure of polymers, including PDEBA. In [41], only two isomers of the model monosubstituted polyacetylene (trans-transoid chain and cis-transoid chain) with different types of connection of links (head or tail) were considered, taking into account possible rotations of links around the C=C bond. The articles devoted to the synthesis of polyDEB almost always consider only the possibility of preferential formation of cis- [11,17,42] or trans- [43,44] isomers concerning the C=C bond of the main chain. However, it is theoretically possible to form four types of conformers (**VII**–**X**), including due to rotation around a single C–C bond. In addition, the difference between the H and R = –PhC≡CH substituents makes it possible to attach monomer units of the type “head-to-tail” (a) or “head-head-tail-tail” (b) for each of the four conformers (Figure 10).

Linear polyene PDEBA molecules are rigid rods for which the concept of segmental mobility is not applicable. Therefore, there are severe restrictions on the steric availability of phenylene and ethynyl fragments in the central links of macromolecules. To clarify the steric features of synthesized PDEBA, their Stuart–Briegleb molecular models were constructed and studied. It was found that the use of three polymer links makes it possible to assemble a polymer chain of any conformation (Figure 10VII–Xa,b). Therefore, to obtain reliable results, polymer chains were built from 10 or more monomer units. At the same time, we tried to evaluate the steric availability of various substituent –PhC≡CH fragments for modification reactions.

In the cis-S-cisoid structure of **VII** PDEBA, the macromolecule easily forms a helix in both cases: both in the case of the addition of monomers by the head-to-tail type and by the head-to-head type. CH≡ and C≡C fragments will be able to form acetylides, carboranes, π-acetylene complexes with mononuclear carbonyls and Co_2_(CO)_8_. The possibility of the formation of a π-arene mononuclear complex between Me(CO)_6_ and -Ph- is not excluded. The reaction of Co_2_(CO)_8_ with the -Ph- group is excluded.

The cis-S-transoid structure is not realized as a strict conformer **VIII** in any way of linking units. Even with significant rotation of the macromolecule around the C–C bond, the model is practically not assembled. The spatial chaotic arrangement of the –PhC≡CH substituents demonstrates that it is practically impossible to add monomers to the macrochain during its formation during polymerization. That is, DEB polymerization cannot occur with the formation of a cis-S-transoid structure.

In isotactic trans-S-trans structure **IXa** –PhC≡CH substituents are tightly stacked along the macromolecule axis. The planes of the –Ph– groups deviate from the planes orthogonal to the macromolecule axis by an angle of up to ±10–20°. The macromolecule is twisted along the axis with a return period of 18–22 links. The C≡C groups are practically inaccessible for both metal carbonyls and decaborane. Only the –Ph– and –C≡CH groups of the terminal units of macromolecules are available. Replacement of acidic hydrogen in –C≡CH groups with copper is possible. However, this requires a stronger rotation of the macromolecule along its axis because of the significantly larger diameter of the Cu atom as compared to the diameter of the hydrogen atom in the terminal ethynyl HC≡ group.

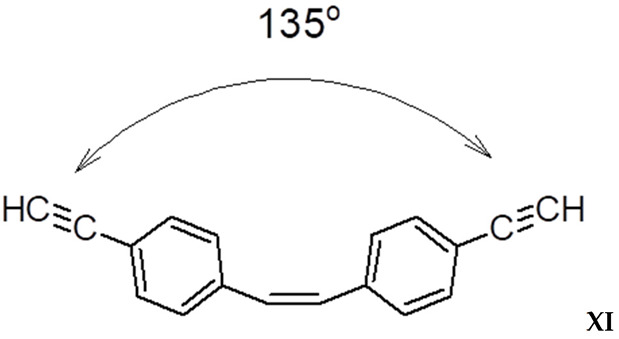



In the syndiotactic trans-S-trans structure **IXb** (head-to-head) –PhC≡CH substituents are stacked in two less dense stacks along a linear macromolecule. In this case, the axes of these substituents are perpendicular to the axis of the macromolecule. A macromolecule can lie on a plane. The planes of the –PhC≡CH substituents can freely deviate from the orthogonal plane by an angle up to ±40–50°. The C≡CH groups can form σ-acetylides, carborane, and π-complexes. Ph fragments can form a π-arene single-core carbonyl only under the condition of maximum deviation of the planes of neighboring -PhC≡CH groups in opposite directions from orthogonality, which allows the constructed model. In addition, syndiotactic trans-S-trans PDEBA (head-to-head) can take the form of a spiral **XI** (end view). At the same time, ≡CH, C≡C and –Ph fragments become more accessible for the synthesis of σ-acetylides, carborane, and a π-arene single-core complex.

The trans-S-cisoid structure of PDEBA can be realized only in the case of joining links of the head-to-tail type **Xa**. The macromolecule cannot twist along its axis. –PhC≡CH substituents form stacks on different sides of the macromolecule. The angle between the stacks is 130–135°, as in the case of model **XI**. The planes of the –PhC≡CH substituents can deviate by an angle up to ±10° from the orthogonality relative to the axis of the macromolecule. Visually, the macromolecule resembles a wide ribbon. CH≡ and C≡C fragments can form acetylides, carboranes, one- and two-core π-carbonyl complexes. Ph fragments cannot form complexes.

Thus, taking into account the results of the construction of Stuart-Briegleb models, it should be assumed that during polymerization of DEB:

- formation of cis-S-transoid structure **VIII** is not possible;
- the trans-S-cisoid structure cannot be realized when connecting the head-head-tail-tail links **Xb**;
- other types of structures **VII**, **IX**, **Xa** may be formed.

### 3.5. Steric Features of DEB-DPDA Copolymers Molecules

Anionic statistical CPA represent substituted polyenes with alternating –H, –PhC≡CH, –C≡CPh and –Ph substituents. The nature of the alternation of these substituents will depend on the following factors:

- the ratio of monomers in the copolymers;
- a different combination of two types of attachment (head-to-tail, head-to-head) for each of the substituents.

In turn, the intramolecular structure of CPA will affect the possibility of modification reactions taking into account the steric capabilities of macromolecules. The copolymerization constants of comonomers do not differ significantly, so the main features of the CPA conformations will be considered for simplification using examples of the theoretically most likely molecular models of alternating copolymers **XII**–**XVII**.

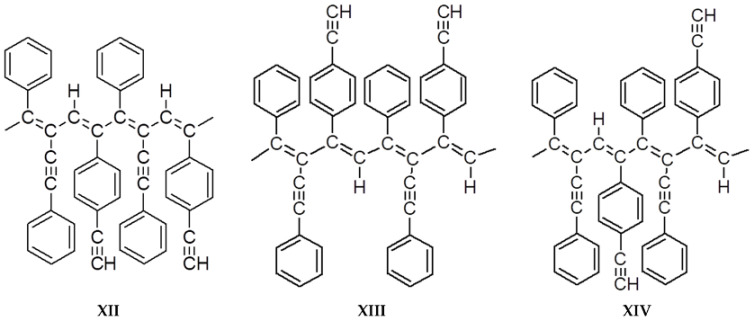

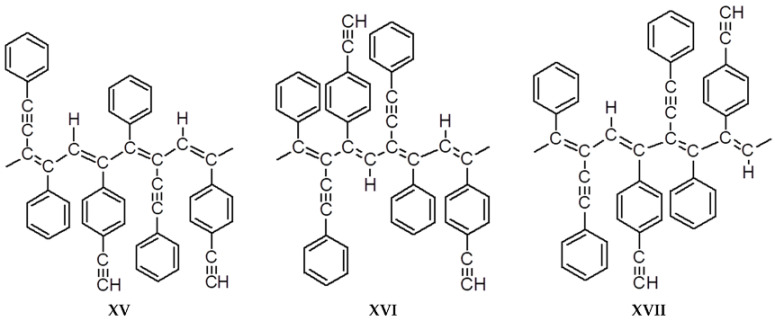





When considering all types of conformations of copolymers, it was found that substituents are always arranged in stacks of different densities along the main chain, regardless of the ratio of monomers and the type of connection of the links. The main polymer chain is always twisted along its axis with a period depending on the structure of a particular copolymer.

In structure **XII**, the polyene chain is twisted with a repeatability period through 20–22 links. The –Ph substituents of the polyene chain may deviate from orthogonality by ±35–45° and are not available for metal carbonyls. Ph- fragments in substituents of –C≡CPh deviate from orthogonality by an angle up to ±30° and are also sterically inaccessible. All C≡C groups cannot form π-complexes or carboranes. The formation of an organometallic compound ≡CCu is possible.

Macromolecule **XIII** makes a complete turnover with a period of 22–24 links. The -Ph- fragments and -Ph substituents of the polymer chain can simultaneously deviate from orthogonality by an angle up to ±20–30°. It is possible to simultaneously rotate the plane of the –C≡CPh substituents in one direction by an angle of up to ±60°. However, the magnitude of this angle can reach a limit value of 90° for individual substituents of the –C≡CPh, if the planes of the –C≡CPh substituents adjacent to them remain orthogonal to the axis of the macromolecule. Thus, aromatic -Ph- and Ph-fragments as well as C≡C groups in –C≡CPh are sterically inaccessible to the reagents used. On the contrary, the HC≡C groups can form acetylides, carbonyl π-complexes, and carboranes.

In the **XIV** structure, the macromolecule is twisted along its axis with a period of 12–14 links. All aromatic fragments deviate from orthogonality by an angle up to ±25–30° and are sterically inaccessible for the formation of complexes with metal carbonyls. All ≡CH groups can form copper acetylides. The C≡C groups in the –C≡CPh substituents, as well as in the -PhC≡CH substituents located between these substituents, are not available for the formation of π-complexes or carboranes. C≡C groups in -PhC≡CH, located on the other side of the macromolecule in every fourth link, can form π-complexes and carboranes.

The macromolecule **XV** is twisted along the axis with a turnover of 12–14 links. The planes of the Ph-, –C≡CPh and –PhC≡CH substituents located next to one side of the macromolecule (the lower part of model **XV**), deviate from orthogonality by an angle up to ±25–30°. Ethynyl and aromatic fragments in them are inaccessible to the formation of π-complexes and carboranes. The Ph- substituents adjacent to the H substituents in the upper part of the **XV** model may deviate from the orthogonality by an angle up to ±35–45° but nevertheless remain inaccessible to metal carbonyls. The planes of the –C≡CPh substituents adjacent to the H substituents of in the upper part of the molecule are capable of deviating from orthogonality by a limiting angle of ±90°. Therefore, it is possible for these fragments to form π-arene, π-acetylene complexes, and carboranes with the –C≡CPh substituents. The **XV** structure allows the synthesis of Cu acetylide.

Structures **XVI** and **XVII** are macromolecules twisted along the axis with a repeatability period of 16–18 links. The planes of the substituents can deviate from the orthogonality in each of the sides by an angle up to ±25–30°. With the use of CPA having this structure, Cu acetylides can be synthesized. It is possible, although difficult, the formation of π-acetylene carbonyl and π-arene complexes by both types of fragments:

- C≡C for –C≡CPh and –PhC≡CH substituents;
–Ph for –C≡CPh substituents

The formation of a π-complex between Co_2_(CO)_8_ and C≡C fragments can be carried out provided that the macromolecule is more twisted along its axis, which is quite acceptable.

It should be noted that the assumptions about the possibility of synthesizing various PDEBA derivatives are very conditional and require a more accurate assessment. For example, the size of a Cu atom is more than five times the size of the H atom [45].

Therefore, carrying out the modification reaction will probably require stronger twisting of the PDEBA or CPA chains to ensure the steric availability of the –PhC≡CH substituents. Stuart-Briegleb models demonstrate this capability. Based on the real possibility of the formation of five variants of various cis-trans isomers in the chains of anionic homo- and copolymers (**VII**, **IX**, **Xa**), in our opinion, thorough spectral studies of the synthesized polymers are necessary for the final and reliable solution of the structural problem. These results will determine the accuracy of prediction about the possibility of carrying out any modification reactions of anionic homo- and copolymers.

### 3.6. Modified Polymers PDEBA

The introduction of heteroatoms into poly-conjugated polymers leads to the appearance of new interesting properties of these polymers. Metal-containing polymers have photo- and electrocatalytic, magnetic, optical, electrical, photovoltaic and electrochemical properties [46,47]. PDEBA has reactive –PhC≡CH groups. Based on a linear PDEBA having reactive –PhC≡CH groups, copper σ-acetylides, its carborane derivative, and dicobalt hexacarbonyl π-complexes were synthesized for the first time [39,40]. The first results are given in the following sections.

#### 3.6.1. Synthesis of Dicobalt Hexacarbonyl π-Complexes

**Scheme 3:**
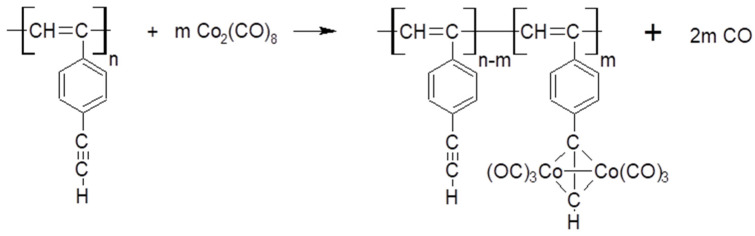
Scheme of synthesis of dicobalt hexacarbonyl π-complexes PDEBA.

There are known syntheses of Co_2_(CO)_8_ complexes with ethynyl and ethynylene groups of highly branched [48,49] and ethynylene groups of linear polyconjugated polymers [50]. We first synthesized the complex according to Scheme 3 [38] using weakly branched PDEBA-3.

The resulting complexes were paramagnetic brown powders insoluble in organic solvents. It should be emphasized that the complexes lost solubility after sedimentation. At the maximum ratio –C≡CH:Co_2_(CO)_8_ = 1:1 (Table 7), an insoluble product was formed already in the synthesis phase. Earlier [34] it was shown that under mild conditions under which the complex is obtained (room temperature), there is no opening of C≡C bonds in the monomer followed by the formation of macromolecules. Therefore, the loss of solubility during polymer complexation cannot be explained by crosslinking of macromolecules by C≡C bonds. Probably, the formation of more complex inter-chain π-carbonyl complexes occurs, for example, Co_4_(CO)_12_(C≡CR)_2_ [51].

The formation of Co_2_(CO)_6_↔C≡C fragments was confirmed by IR spectroscopy. In the region of 2100–2000 cm^−1^ of the IRS of the benzene solution PDEBA–Co–1, four specific peaks of high intensity of valence vibrations of Co-groups appeared [52]. Bands of valence vibrations of the Co–C bond and deformation vibrations of carbonyl groups were observed in the region of 550–480 cm^−1^. This demonstrated the stability of complexes that do not collapse during polymer sedimentation. The intense band of 3300 cm^−1^ valence vibrations of unreacted ethynyl groups was well manifested [27]. The intensity of this band naturally decreased during the transition from PDEBA–Co–1 to PDEBA–Co–4 due to an increase in the carbonyl content in polymer complexes. The band of 2100 cm^−1^ valence vibrations of the RC≡CH bond also decreased with an increase in the amount of added carbonyl and disappeared in PDEBA–Co–4. Valence vibrations of a monosubstituted triple bond, π-coordinated with cobalt carbonyl, give a peak of 1550 cm^−1^ [52]. However, this band could not be detected, because there are intense signals of valence vibrations of the benzene ring in this region. The formation of π-aromatic complexes between phenyl nuclei and cobalt carbonyl did not occur, since no changes in the bands characteristic of phenylene groups were observed.

#### 3.6.2. Copper σ-Acetylides of Poly-*p*-Diethynylbenzene

A large number of low-molecular-weight copper σ-acetylides with the general formula R–C≡C–Cu are known [53,54], however, polymer copper σ-acetylides are unknown. The interest in the synthesis of linear PDEBA copper σ-acetylide was caused by the information that low molecular weight copper σ-phenylacetylide has photoelectric sensitivity [55]. However, the crystalline nature of copper phenylacetylide did not allow obtaining thin homogeneous photosensitive materials even when creating composite materials such as polymer matrix—copper acetylide. We formulated the goal: is it possible to synthesize polymeric copper acetylide based on linear soluble PDEBA? The polymer base, in principle, can allow:

- to obtain a soluble copper-containing polymer material capable of forming films;
- create an extended cluster of copper atoms.

**Scheme 4:**
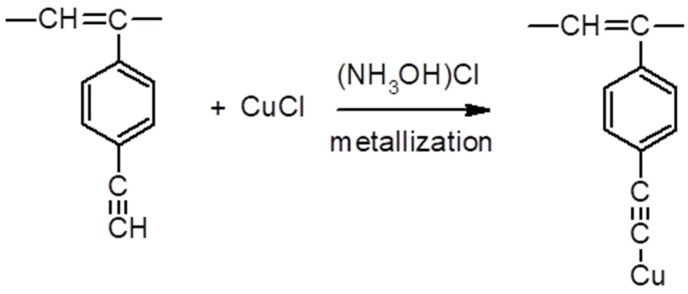
Scheme of synthesis of polymeric σ-copper acetylide.

To synthesize polymeric σ-copper acetylides in accordance with Scheme 4, we used PDEBA-1, which has an ethynyl group in each unit of the macromolecule.

Unfortunately, the obtained polymeric copper σ-acetylides (PDEBA-Cu) were brown powders insoluble in organic solvents. The elemental analysis showed (Table 8) that links appear in the modified polymer, the structure of which can be described by the formula –[CH=C(Ph–C≡C–Cu)]_n_–. However, in an experiment with the ratio of [Cu^+^]_o_/[C≡CH]_o_ = 3/10, the formation of –C≡CCu fragments turned out to be small. A possible reason was the large size of the Cu^+^ ion. This could allow it to join the –C≡CH groups only of the end links of the polymer chain due to steric obstacles.

The presence of acetylide units in the PDEBA-Cu was confirmed by the appearance in the IR spectrum of the modified polymer of the 1920 cm^−1^ band of valence vibrations of the C≡C bond associated with the copper atom. A similar band is given by the triple bond PhC≡CCu in copper phenylacetylide [53].

The lack of solubility of PDEBA-Cu could be explained by the crosslinking of macromolecules occurring during the interaction of ethynyl groups with Cu^2+^ with the formation of inter-chain –Ph–C≡C–Cu–C≡C–Ph– fragments as proposed in [56]. At the same time, the Cu^2+^ species could be present as an impurity in CuCl. However, it was determined by the XPS that copper in σ-acetylide PDEBA was present in the form of Cu^+^. The binding energy of Cu 2p_3/2_ peak of the polymer σ-acetylide is close to that of the model copper phenylacetylide (933.4 and 933.2 eV, respectively) and literature data [57,58].

This proved the presence in the modified PDEBA of only C≡C–Cu fragments.

**Scheme 5:**
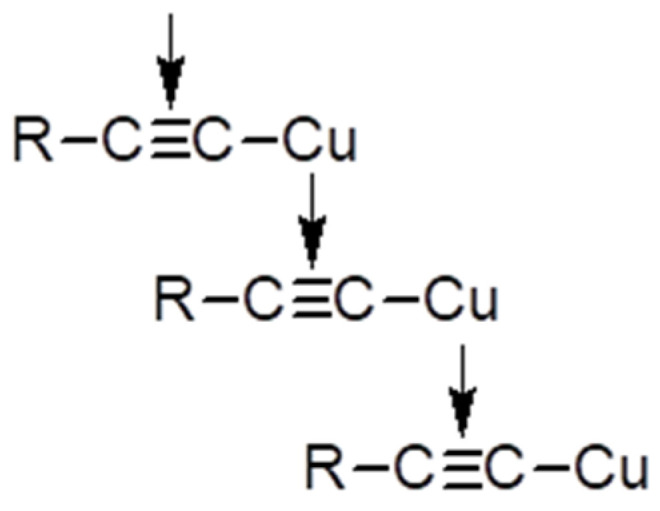
Formation of coordination acetylides.

The existence of Cu^+^ acetylides in the form of so-called “coordination polymers” with varying degrees of association is described in [59,60,61]. Coordination of acetylides is carried out due to the formation of complex bonds of copper atoms with ethynyl groups according to Scheme 5.

Thus, the insolubility of Cu polymer σ-acetylides can be explained by the formation of such coordination bonds. The beginning of the formation of a cross-linked structure already during the synthesis of PDEBA-Cu could be an additional reason for the low copper content in the polymer due to the deterioration of the steric availability of the –C≡CH groups in the polymer grid. In this case, two processes (the formation of a polymer mesh and the diffusion of Cu^+^ ions) can compete.

#### 3.6.3. Carborane-Containing PDEBA and CPA

The reaction of decaborane with low molecular weight acetylenes is well known [62]. This reaction was used to create polymer carborane-containing clusters using poly-conjugated ethynylene-containing polymers [63,64], as well as for the creation of carboranes in the side hangers of polystyrene [63]. The synthesis of various boron-containing polymers is due to the creation of luminescent, electroactive, sensory, thermo-, and fire-resistant materials [63,64,65]. The reaction of decaborane modification of a polymer containing –C≡CH groups and synthesized in the presence of a Ni(C_5_H_7_O_2_)_2_/Ph_3_P complex is described [66]. However, doubts arise about the correctness of the description of the intramolecular structure of the resulting substance, since there are contradictions in the interpretation by various authors of the intramolecular structure of the initial polyaryl acetylene resin. The authors [66,67] believe that the poly-DEB has a mostly linear structure. On the other hand, [68,69] indicates the branched structure of polyDEB with the presence of even phenylene fragments in them.

**Scheme 6:**
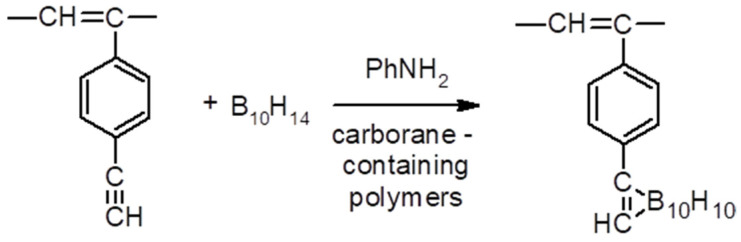
Formation of o-carborane-containing links.

For modification, we used PDEBA-1 and CPA-3. O-carborane-containing polymers (PDEBA-1B and CPA-3B respectively) were synthesized according to Scheme 6.

PDEBA—1B1 (20.4% boron), PDEBA-1B2 (35.2%) and CPA-3B (26.5% boron) were yellow-brown powders, highly soluble in benzene and HMPA. A wide band at 2570 cm^−1^ (valence vibrations of the B-H bond [62]) was detected in the IR spectra of the obtained carborane-containing products. Absorption in the region at 3300 cm^−1^ (valence vibrations of the ≡C–H bond) decreased in PDEBA-1B2 or disappeared completely in CPA-3B.

**Figure 11:**
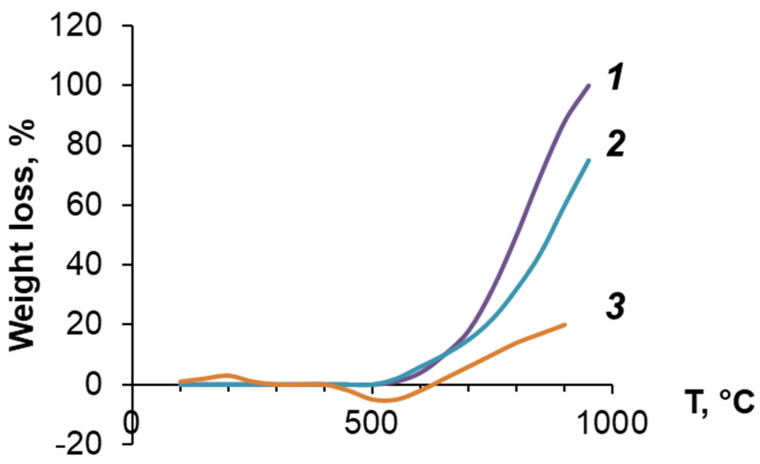
TGA curves, air: (**1**)—PDEBA-1; (**2**)—PDEBA-1 in a vacuum; (**3**)—PDEBA-1B1.

**Figure 12:**
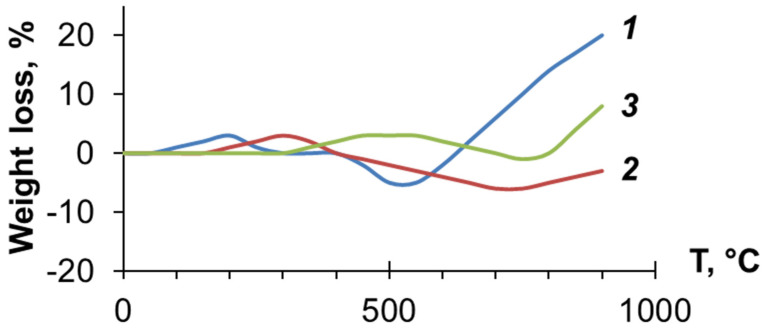
TGA curves in the air for PDEBA-1B1 (**1**); PDEBA-1B2 (**2**); CPA-B (**3**).

There was no noticeable pursuit of the mass of the original PDEBA-1 when it was heated in the air to 400 °C (Figure 11, curve 1). Further, the rate of thermal degradation increased, and at 625 °C the mass loss was 80%. A significant change in thermal-oxidative degradation was observed for PDEBA-1B1. At 920 °C, the mass criterion of PDEBA-1B1 was only 22% (Figure 12, curve 4). Thus, modified boron polymers have acquired a higher resistance to thermal-oxidative degradation.

**Figure 13:**
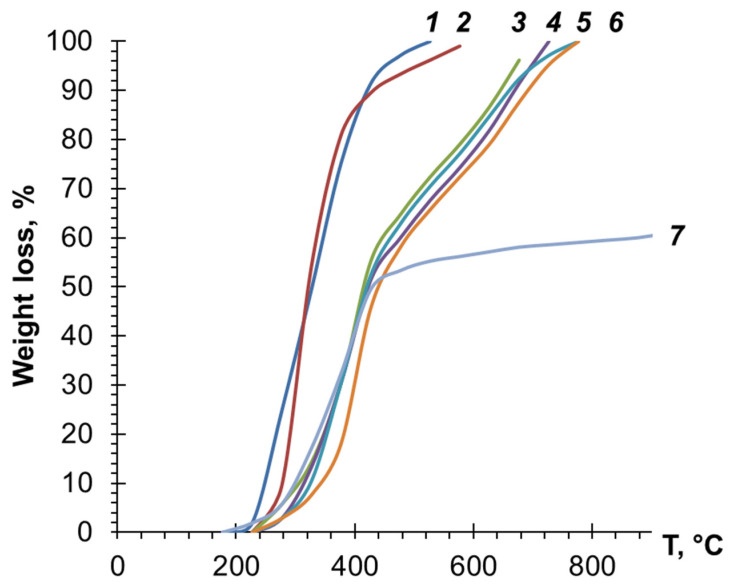
TGA curves of compounds in air: (**1**) TGM-3, (**2**) OCM-2, (**3**) TGM-3 (80%) + PDEBA-6 (20%), (**4**) TGM-3 (64%) + PDEBA-6 (36%), (**5**) OCM-2 (68%) + PDEBA-6 (32%), (**6**) TGM-3 (68%) + PDEBA-6 (32%), and (**7**) TGM-3 (72%) + PDEBA-6B1 (28%).

Figure 13 shows the comparative results of TGA in the air of synthesized boron-containing polymers. PDEBA-1B1 and CPA-3B at a temperature of 900 °C lost 19 and 8% of the mass, respectively. A more significant weight loss in PDEBA-1B1 compared to CPA-3B can be explained by the presence of ethynyl groups in PDEBA-1B1 (42% of the initial amount), which were easily subjected to thermal-oxidative degradation. At the same time, in CPA-3B (according to IR spectroscopy), all ethynyl groups –C≡CH reacted with decaborane to form an o-carborane core. The increase in the mass of samples in the processes of thermo-oxidative destruction of all samples can be explained by the formation of oxygen-containing boron compounds [70]. The study of the thermal-oxidative destruction of the carborane-containing homopolymer PDEBA-1B2, which does not have ethynyl groups, showed that it has no mass loss at a temperature of 900 °C (Figure 12, curve 2). Moreover, the mass of the sample increased due to the addition of oxygen with the formation of oxygen-containing boron compounds.

### 3.7. Polymers of DEB as an Industrial Material Modifier

We evaluated the possibility of using DEB-containing polymers for the modification of some industrial materials.

#### 3.7.1. Modification of Industrial Oligoester Acrylates

The research of oligoester acrylates (OEA) is explained by their wide use as various materials [71,72,73,74]. However, OEA does not have high-temperature resistance to solve some technical problems. Therefore, a study was made on the effect of PDEBA-6 and PDEBA-6B1 additives on the thermal oxidative stability of two industrial OEA:

- TGM-3 CH_2_=C(CH_3_)–C(O)–(OCH_2_CH_2_)_3_–O–C(O)–C(CH_3_)=CH_2_
- OCM-2 CH_2_=C(CH_3_)–C(O)–OCH_2_CH_2_OC(O)–(CH_2_CH_2_O)_2_C(O)OCH_2_CH_2_O–C(O)C(CH_3_)=CH_2_

To do this, 20–36% PDEBA-6 and PDEBA-6B1 were added to the OEA. It was found that:

- the heat resistance on the air of both cured oligoesteracrylates increases significantly with the addition of PDEBA-6;
- the viscosity of compositions with PDEBA-6 increased significantly with the addition of ≥32% PDEBA-6;
- the heat resistance of the cured oligoesteracrylates increased with an increase in the amount of PDEBA-6 added;
- the decrease in mass loss of compositions with PDEBA-6 compared to cross-linked OEA was most significant at the highest temperatures;
- in the case of using PDEBA-6B1, the mass loss of TGM-3 even >1200 °C was less than 60%.

Thus, a significant increase in the thermal-oxidative stability of OEA grids in the presence of PDEBA-6 was found (curves 3–6 in Figure 13) and, especially, PDEBA-6B1 (curve 7).

#### 3.7.2. Modification of Industrial Epoxy Novolac Resin

To create carbon-carbon composite materials, initial epoxy-containing and phenol-formaldehyde resins are used, which are subjected to carbonation [75,76]. The technological process of pyrolysis of the initial composite is more efficient and economical in the case when the coke residue is large. Therefore, an important task was to develop new methods for the preparation of a carbon matrix in order to increase the amount of coke residue, for example, by mixing phenol-formaldehyde resin and resin obtained by cyclotrimerization of diethynylbenzene [75]. In this paper, the process of modification of the widely used epoxy novolac resin EN-6 using a copolymer CPA-5 and a homopolymer poly-DPDA (for comparison) was investigated. The resin was modified by mixing the components through solvents (acetone, furfural) and melts. The modified resin was cured (250 °C; 2 h), heated in an atmosphere of N_2_ (900 °C; 10 min) and the amount of coke residue was determined. The introduction of 10–30% poly-DPDA into the resin increased coke by 15–21% (Table 9). The introduction of 10–30% CPA-5 gave a greater effect and increased coke by 26–33%. Apparently, this is due to the presence in the copolymer macromolecules of a significant number of reactive C≡CH groups, contributing to a more efficient formation of a three-dimensional grid during pyrolysis of the cured resin.

Thus, the addition of poly-conjugated polymers significantly (by 15–33%) increased the coke residue during pyrolysis of modified industrial resin EN-6. The effect was enhanced by the presence of CPA-5 groups in the modifying –C≡CH additive.

#### 3.7.3. Modification of Oriented Carbon Fibers

The behavior of boron-containing compounds is of practical interest in the synthesis of various carbon materials from the point of view of creating the necessary intramolecular structure of these materials [77,78,79,80].

Earlier [81], the fact of increasing the electrical conductivity of oriented carbon fibers (OCF) using a PDEBA-based composition was discovered. This article discusses in more detail the features that occur during the modification of the OCF in the presence of PDEBA-6 and a carborane-containing oligomer obtained by the interaction of epichlorohydrin with 1.7-bis(oxymethyl)-m-carborane. The initial OCF were impregnated with a solution of a composition of PDEBA-6 (5%) and a carborane-containing oligomer (5%) in THF. The samples were dried in air and pyrolyzed in medium N_2_ at 500–2400 °C. The surface layer with a thickness of 2 nm was studied using XPS. The binding energies of the B 1s and N 1s peaks and their relative contents were determined. From the results obtained, it follows that during the heat treatment of the OCF modified with a carborane-containing composition, BN is formed on its surface as a result of the interaction of the nitrogen of the medium with the carborane core decaying during heating. The highest relative content of boron and nitrogen on the sample surface was observed after treatment at 1500 °C. With further heating, the BN content on the surface decreased markedly (Table 10). Heating above 1500 °C led to the destruction of BN and the migration of boron into the material, apparently in the form of boron carbide [80].

**Figure 14:**
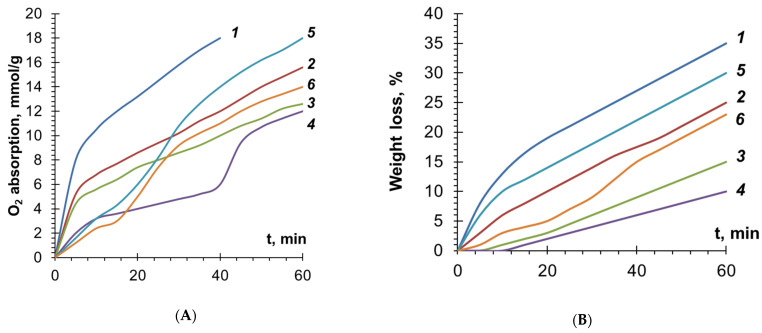
Dependence of the quantity of absorption oxygen (**A**) and thermal-oxidative stability (**B**) of OCF on the temperature of its modification: (**1**)—500 °C; (**2**)—1000 °C; (**3**)—1250 °C; (4)—1500 °C; (**5**)—2000 °C; (**6**)—2400 °C.

For all samples, thermal oxidation stability (oxygen pressure 20 kPa) was determined by thermovolumetry [21] on the kinetics of O_2_ absorption and measurement of the mass loss of the sample at 600 °C for 60 min. The highest thermal oxidation stability was observed for samples also treated at 1500 °C (Figure 14). Apparently, this is due to the graphitization of OCF samples due to the inward migration of boron, which is an effective catalyst for graphitization [77,79,84].

Thus, in the process of heat treatment, thermal decomposition of boron-containing polymer compositions occurs with the formation of intermediate structures and further formation of nitride-boron structures. The disappearance of the latter upon further heating above 1500 °C is accompanied by the diffusion of boron into the material, which should lead to a significant ordering of the carbon structure. This is naturally confirmed by the results of measuring the electrical resistance of the modified material (Table 11). High resistance value after processing at a temperature of 500 °C is explained by the presence on the surface of an amorphous disordered modifying layer with increased electrical resistance. A significant decrease in the electrical resistivity of the OCF at a temperature > 1500 °C confirms the assumption made about the nature of the ongoing processes.

Indeed, a comparison of XRD patterns of different samples showed a significant improvement in supramolecular packing in oriented carbon with an increase in the temperature of carbon heat treatment. This is evidenced by the appearance of additional long-range peaks on OCF XRD patterns, as well as their narrowing. Thus, the proposed modification of carbon fiber made it possible to give it the necessary good operational bifunctionality: high resistance to oxygen and high conductivity [85,86].

## 4. Conclusions

In the presence of *n*-BuLi, widely used in industry, anionic polymerization of bifunctional DEB and its copolymerization with DPDA were carried out in polar solvents. As a result, completely soluble polymers were formed with conversions of 63 and 65%, respectively, and an ${\bar{M}}_{n}$ value of up to 3730. The intramolecular structure of the synthesized polymers was determined using IRS, ^1^H, ^13^C NMRS, and GPC. It has been proven that the authors synthesized for the first time a completely linear PDEB with side substituents –PhC≡CH in the HMPA medium. The use of DMSO leads to the appearance of lateral branches. The transfer of the chain to the polymer during polymerization was detected. High thermo- and thermo-oxidative stabilities of polymers have been demonstrated. Macromolecular acetylides, carboranes, and arencarbonyl π-complexes can be synthesized using side reactive –PhC≡CH groups present in polymers. Using Stuart-Briegleb models, the possibility of implementing various polymer stereoisomers has been analyzed. It is shown that there are isomers that cannot be realized during polymerization. In addition, not for all real stereoisomers, it is possible to carry out a synthesis reaction of polymer organoelement compounds. Despite the low value of ${\bar{M}}_{n}$, *p*-diethynylbenzene polymers have been successfully used to modify commercial samples of novolac epoxy resin, oligoetheracrylates, and carbon fibers.

## Figures and Tables

**Table 1:** Polymerization conditions of the DEB, conversion degree Y and properties of obtained polymers (initiator *n*-BuLi; 55 °C; [*M*]_0_/[*I*]_0_ = 15:1; [*M*]_0_ = 0.7 mol L^−1^).

Samples	Solvent	τ, Min	Y, %		${\bar{M}}_{n}/n{\;}^{c}$	H_ar+ol_/H_eth_ ^d^
			Sol ^a^	Ins ^b^		
PDEBA-1	HMPA	5	47	-	1300/10.3	5/1
PDEBA-2	HMPA	20	48	-	1340/10.6	5/1
PDEBA-3	HMPA	40	49	-	1370/10.9	5.3/1
PDEBA-4	DMSO	1	5	-	1800/14	7/1
PDEBA-5	DMSO	20	58	23	3160/25	7/1
PDEBA-6	DMSO	60	63	26	3730/29.6	8/1

^a^ is the fraction soluble in organic solvents. ^b^ is the fraction insoluble in organic solvents. ^c^ is the value calculated for the fraction soluble in organic solvents. ^d^ is the ratio of the sum of the integral intensities of signals of aromatic and olefin protons to the integral intensity of signals of protons of ethynyl groups in ^1^H NMR spectra of samples.

**Table 2:** Comparison of theoretical ${\bar{n}}_{theor}$ and experimental ${\bar{n}}_{exp}$ values of the number average degree of polymerization.

Degree of Polymerization	Polymer		
	PDEBA-1	PDEBA-2	PDEBA-3
${\bar{n}}_{theor}$	7.05	7.20	7.26
${\bar{n}}_{exp}$	10.32	10.63	10.87

**Table 4:** Parameters of molecular mass distribution of PDEBA.

Samples	${\bar{M}}_{n}$	${\bar{M}}_{w}$	${\bar{M}}_{z}$	${\bar{M}}_{w}/{\bar{M}}_{n}$	${\bar{M}}_{z}/{\bar{M}}_{w}$
PDEBA-1	1300	2380	7200	1.82	3.02
PDEBA-2	1340	2430	7300	1.82	3.01
PDEBA-3	1370	2700	8700	1.97	3.20
PDEBA-4 ^a^	1800	2400	4400	1.33	1.83
PDEBA-5 ^a^	3160	5400	14,000	1.70	2.66
PDEBA-6 ^a^	3730	10,800	24,000	2.89	2.22

^a^ For the soluble in benzene fraction.

**Table 5:** Influence of monomers mixture composition on the conversion Y and properties CPA (initiator *n*-BuLi, solvent DMSO, [*M*]_0_:[*I*]_0_ = 15, T = 55 °C, [*M*]_0_ = 0.7 mol·L^−1^).

Samples	Initial Ratio $\frac{{\left[DEB\right]}_{o}}{{\left[DPDA\right]}_{o}}$	τ, Min	Y, %	${\bar{M}}_{n}$	$\frac{H_{eth}}{H_{ar+ol}}{\;}^{a}$	–C≡CH, % Mass ^b^	Quantity –C≡CH in a Macromolecule
CPA-1	1:3	1	3	860	1:31	3.3	1.1
CPA-2	1:3	60	41	1070	1:40	3.0	1.3
CPA-3	1:1	1	4	1650	1:18	6.3	4.2
CPA-4	1:1	60	30	1940	1:21	5.7	4.5
CPA-5	3:1	1	5	1500	1:11	10.2	6.1
CPA-6	3:1	60	65	1890	1:13	9.4	7.1

^a^ *H_eth_*:*H_ar+ol_* is the ratio of the integral intensity of the signals of ethynyl protons to the sum of the integral intensities of the signals of aromatic and olefinic protons in the ^1^H NMR spectra; ^b^ Quantity of incorporations of ethynyl groups in CPA was calculated by the formula $\%C\equiv \mathrm{CH}=\frac{H_{eth}\cdot MM_{C\equiv CH}}{H_{eth}\cdot MM_{DEB}+0.1\left(H_{ar}-5H_{eth}\right)}:\frac{1}{MM_{DPDA}}$, where *H_eth_*, *H_ar_*, *MM_DEB_*, and *MM_DPDA_* are the molecular weights of the ethynyl group, aryl group, DEB, and DPDA, respectively.

**Table 6:** Dependence of the yield Y and the composition of CPA on the composition of the monomer mixture. Parameters for calculating copolymerization constants according to the Fineman and Ross equation.

Samples ^a^	$\frac{{\left[DEB\right]}_{o}}{{\left[DPDA\right]}_{o}}$ ^b^	Y, %	$\frac{H_{eth}}{H_{ar+ol}}$ ^c^	Ratio $\frac{\left[DEB\right]}{\left[DPDA\right]}\;$ in CPA	$F=\frac{{\left[DEB\right]}_{o}}{\begin{array}{c} {\left[DPDA\right]}_{o} \end{array}}$	$f=\frac{\left[DEB\right]}{\left[DPDA\right]}$	$\frac{F^{2}}{f}$	$\frac{F\left(f-1\right)}{f}$
CPA-1	1:3	3.5	1:31.0	1:1.80	0.33	0.556	0.200	−0.267
CPA-7	1:3	3.5	1:34.0	1:2.16	0.33	0.463	0.240	−0.387
CPA-8	1:3	3.0	1:35.8	1:2.28	0.33	0.439	0.253	−0.427
CPA-9	1:2	4.2	1:25.3	1:1.37	0.50	0.730	0.343	−0.185
CPA-3	1:1	3.8	1:17.6	1:0.80	1.00	1.250	0.800	0.200
CPA-5	3:1	4.6	1:11.0	1:0.29	3.00	3.448	2.610	2.130

^a^ Copolymerization time 1 min. ^b^ Initial ratio of comonomers. ^c^ *H_eth_*:*H_ar+ol_* is the ratio of the integral intensity of the signals of ethynyl protons to the sum of the integral intensities of the signals of aromatic and olefinic protons in the ^1^H NMR spectra.

**Table 7:** Composition of PDEBA π-complexes with Co_2_(CO)_8_.

Samples	Initial Ratio $\frac{{\left[C\equiv CH\right]}_{o}}{{\left[Co_{2}{\left(CO\right)}_{8}\right]}_{o}},$ mol per mol	Reaction Time, Hour	Content Co, % Mass ^a^		Polymer Complex Ratio $\frac{\left[C\equiv CH\right]}{\left[Co_{2}{\left(CO\right)}_{6}↔C\equiv C\right]},\;$ mol per mol
			Theor.	Exp.	
PDEBA-Co-1	3:1	3	14.50	12.55	2.45:1
PDEBA-Co-2	2:1	8	17.32	15.90	1.26:1
PDEBA-Co-3	1.5:1	15	21.50	19.55	0.65:1
PDEBA-Co-4	1:1	20	25.61	24.59	0.04:1

^a^ Elemental analysis results.

**Table 8:** Composition of poly p-DEB copper acetylides.

Initial Ratio: $\frac{\left[Cu^{+}\right]}{\left[-C\equiv CH\right]}$	PDEBA-Cu Polymer Complex Ratio: $\frac{\left[-C\equiv CCu\right]}{\left[-C\equiv CH\right]}$
1:10	1:10.2
3:10	1:11.7

**Table 9:** Effect of polymer additives on the yield of EN-6 resin products.

№	Composition Formulation, % _mass_.			Coke Yield, %
	CPA-5	Poly-DPDA	EN-6	
1	−	−	100	29.95
2	10	−	90	62.80
3	20	−	80	55.84
4	30	−	70	59.61
5	−	10	90	44.91
6	−	20	80	49.66
7	−	30	70	50.81

**Table 10:** Results of OCF analysis.

Sample	Treatment Condition		B ^a^, % _mass_.	XPS Surface Analysis ^b^			
	T, °C	Medium		B		N	
				Binding Energy, eV	Relative Content, %	Binding Energy, eV	Relative Content, at. %
1	500	N_2_	0.63	190.1; 189.1	1	-	-
2	1000	N_2_	0.53	187.7; 186.2	2	399.0	5
3	1250	N_2_	0.48	190.6	10	398.4	30
4	1500	N_2_	0.62	190.9	20	398.7	80
5	2000	N_2_	0.76	190.7	2	398.7	5
6	2400	N_2_	0.30	190.3	Traces	398.5	Traces
7	2400	Ar	0.10	188.5	Traces	-	-

^a^ Elemental analysis. ^b^ For the standards BN, B_4_C, B, and B_2_O_3_, the binding energies of the B 1s peaks are 190.6; 188.1; 187.5; and 193.0 eV, respectively [82,83]. For the BN reference, the binding energy of the N 1s electrons is 398.5 eV [82,83].

**Table 11:** The specific electrical resistance of modified carbon.

Sample Processing Temperature, °C	Specific Electrical Resistance, Ω·mm^2^/m
Without modification	35
500	120
1000	88
1250	41
1500	32
2000	31
2400	7
2400 (argon)	18

## Data Availability

The data presented in this study are available on request from the corresponding author.
